# Modified McKenzie procedure for the treatment of fixed painful torticollis

**DOI:** 10.3171/2020.5.FOCVID205

**Published:** 2020-10-01

**Authors:** Zaid Aljuboori, Tyler Ball, Haring Nauta

**Affiliations:** Department of Neurosurgery, University of Louisville, Kentucky

**Keywords:** pain, torticollis, McKenzie, denervation, selective

## Abstract

Spasmodic torticollis is characterized by involuntary movements of the neck muscles. In this video, the authors present the case of a 48-year-old man with painful right-sided rotational torticollis with contributions from both the suboccipital and the left sternocleidomastoid (SCM) muscles. He underwent a suboccipital craniectomy and C1–2 laminectomy with selective denervation of bilateral suboccipital and left-sided SCM muscles (modified McKenzie procedure). At the 2-week follow-up, he showed significant improvement and was able to rotate his neck about 70° toward the midline. Surgical treatment of spasmodic torticollis focuses on interrupting the motor pathway responsible for head turning. The modified McKenzie procedure is valuable, especially when other therapies fail.

The video can be found here: https://youtu.be/TK-WybKnGJM

## Transcript

The modified McKenzie procedure for the treatment of fixed painful torticollis.

### 1:20 Overview of the Anatomy of the Paraspinal and Suboccipital Muscles^1^

• The semispinalis capitis and splenius capitis. The first originates from the cervical facets and transverse processes and the second from the cervical transverse processes. The occiput is the insertion site for both muscles. They are innervated by the dorsal primary rami of C2 and C3 and act to extend and rotate the head.
• The rectus capitis (major and minor). The major originates from the C2 spinous process and the minor from the C1 posterior tubercle, and the occiput is the insertion site for both muscles. They are innervated by the dorsal primary ramus of C1 and act to extend and rotate the head.
• The superior oblique originates from the C1 transverse process and attaches to the occiput. It’s innervated by the dorsal primary ramus of C1 and results in head extension and lateral flexion.
• The inferior oblique originates from the C2 spinous process and attaches to the C1 transverse process. It’s innervated by the dorsal primary ramus of C1 and results in head rotation.

### 1:32 Treatment Options of Spasmodic Torticollis

There are three main treatment options for spasmodic torticollis. The first is Botox injection of the involved muscles. The second is denervation procedures, which include the McKenzie operation, Bertrand procedure, or selective sternocleidomastoid denervation. The third option is the central procedures, which include bilateral thalamotomy (now archaic) or bilateral globus pallidus (internal segment) deep brain stimulation.

Next, we will briefly discuss the techniques, advantages, and disadvantages of these procedures.^2–6^

• The McKenzie operation:
  » The technique involves bilateral intradural motor root section of C1–3, with or without C4 motor root section on the more affected side, with possible selective denervation of cranial nerve XI.
  » Advantages are maximum effect on muscles at the cervical levels of occiput–C1–2 and effect is permanent.
  » Disadvantages are the need for comfort with microsurgery and the caudal limit is C4 because of ventral primary rami to the phrenic nerve and the brachial plexus. Also, it rarely causes dysphagia.
• The Bertrand procedure:
  » The technique involves an extradural unilateral section of the dorsal primary rami C1–5 branches with selective sternocleidomastoid denervation of the opposite side as a separate operation.
  » Advantages are it’s an extradural procedure, it can extend denervation to more caudal levels than the McKenzie, and can include myectomy.
  » Disadvantages are the potential for muscle reinnervation, extensive intramuscular dissection, and hunt for nerve branches using stimulation. Also, a minimum of two approaches needed on opposite sides.
• DBS:
  » The technique involves insertion of bilateral electrode targeting the GPi.
  » Advantages: It directs therapy closer to causal structure and mechanism. It’s reversible with adjustable stimulation parameters.
  » Disadvantages: It’s a two-stage procedure with follow-up for adjustments which could cause compliance issues. There is a potential for equipment failure and maintenance which raises the issue of cost and compliance. Also, the potential for permanent dysarthria from electrode placement alone.
  • Outcomes: Ravindran et al.^7^ performed a systematic review of DBS versus peripheral denervation for cervical dystonia, and they concluded that:
  » Both DBS and selective peripheral denervation are effective for spasmodic torticollis.
  » DBS is effective at reducing symptom severity and disability but with minimal effect on pain.
  » Though both interventions are associated with clinical advantages and disadvantages, specific subpopulations may preferentially respond to one intervention over the other.

### 4:32 Case Presentation

A 48-year-old man presented with chronic, painful, fixed right-sided rotational torticollis.^8^ His main complaint was pain since he had acclimated over many years to his rotated posture. On physical exam, his head-on-neck position is in full rotation toward the right side and his chin almost touching his right shoulder.

Besides, most of the muscle movement seemed to be from the suboccipital muscles, with some participation of the left sternocleidomastoid muscle, as most spasmodic torticollis patients have two axes of movement involved. Even with force, the patient neck could not be turned to a neutral position.

### 5:03 Treatment Decision

We chose the modified McKenzie procedure because of the patient’s psychiatric problems (history of schizophrenia), preference against any brain implants, compliance issues, and pain-dominant complaints. Besides, he had involvement of both left sternocleidomastoid and bilateral suboccipital/paraspinal muscles. Also, he had one treatment of Botox a year prior with no benefit possibly due to the severity and chronicity of his condition.^4,9^

We thought it was best to provide conceptually simple operation with denervation.^5,6^

Before committing to doing the operation, the patient was examined under general anesthesia with muscle relaxants to prove that his head and neck can be turned into a neutral position allowing the operation. His “fixed” deformity while awake had not yet resulted in fixed contractures at the craniocervical junction.

### 5:26 Description of Procedure

We used SSEP and MEP for neuromonitoring. We also used left sternocleidomastoid and trapezius EMG to perform a selective SCM denervation through selective sectioning of the left spinal accessory nerve.

The patient underwent a midline suboccipital craniectomy with a C1–2 laminectomy. We sacrificed the C1–3 motor rootlets bilaterally with selective denervation of the left sternocleidomastoid through the sacrifice of some of the left spinal accessory nerve rootlets (guided by stimulation).

This figure is a cadaveric dissection that depicts the anatomy of the posterior fossa, cervicomedullary junction, cranial nerves, and related vascular structures (Supplemental Fig. 1).

The patient was put in a prone position with his head fixed in flexion using the Mayfield-Kees system.

Exposure: A midline incision was made to expose the occiput and C1–3 spinous processes; then a subperiosteal dissection was performed on both sides to expose the spinous processes and laminae. A midline suboccipital craniectomy and C1–2 laminectomy were done, and the dura was opened in the midline. The arachnoid was opened sharply, and the leaflets were tacked to the dura using AVM clips.

### 6:36 Demonstration of the Neurovascular Anatomy of the Dorsal Surface Craniocervical Junction

Here you can see the intraoperative anatomy with labeling of the orientation, the right posterior inferior cerebellar artery, the spinal accessory nerve, and the C2 dorsal rootlets.

### 6:47 Demonstration of the Sacrifice of the Left C1 Motor Nerve Root

Next, we expose the right C1 motor nerve root by cutting the dentate ligament. Then we sacrifice the nerve root using bipolar cautery and scissors.

### 6:56 Demonstration of the Identification and Sacrifice of the C2 Ventral Motor Rootlets

Next, we separate the right C2 dorsal sensory rootlets to expose the ventral motor rootlets. Then we sacrifice the motor rootlets using bipolar cautery and scissors.

Similar steps were done to sacrifice the left C1 and C2 motor rootlets as well as the bilateral C3 motor rootlets. As for the left spinal accessory nerve, we used nerve stimulation set to 0.1 mA to identify and sacrifice the motor rootlets that only supplied the left sternocleidomastoid muscle.

### 7:22 Demonstration of the Dural Closure Technique

Closure: The subarachnoid space was irrigated with saline to wash out any residual blood products; then the dura and the arachnoid were closed as a single layer in a running fashion. Then the incision was closed in a multilayered fashion.

Postoperative course: The patient remained neurologically stable with some improvement of his torticollis. At 2 weeks’ follow-up, he showed significant improvement and he was able to rotate his neck to about 70° toward the midline. The left sternocleidomastoid muscle showed a reduction in tone and size, so where the bilateral suboccipital muscles.

Pearls and pitfalls: It’s crucial to examine the patient under general anesthesia with muscle relaxants preoperatively, to prove that the patient head and neck can be turned into a neutral position allowing the operation. Also, it’s important to perform the suboccipital craniectomy part of the procedure to allow exposure of the C1 motor root. Care must be taken not to injure the vertebral artery, PICA, or the spinal cord. It’s important to recognize the nerve of McKenzie, which is a connection between the C1 motor root and spinal accessory nerve. It exists in some patients and, if overlooked, may result in continued symptoms.

In conclusion, several forms of therapy have been described to treat spasmodic torticollis with various success rates. Most surgical procedures focus on interrupting the motor pathway responsible for head turning. Selective denervation of the suboccipital muscles bilaterally and the sternocleidomastoid unilaterally proved to be valuable, especially in patients who failed other therapies or with poor compliance.

## Author Contributions

Primary surgeon: Aljuboori, Nauta. Editing and drafting the video and abstract: Aljuboori, Ball. Critically revising the work: Aljuboori. Reviewed submitted version of the work: Aljuboori, Ball. Supervision: Nauta.

## Supplemental Information

### Online-Only Content

Supplemental material is available online.

*Supplemental Fig. 1*. https://thejns.org/doi/suppl/10.3171/2020.5.FOCVID205.

## Supplemental Information

Supplemental Figure
