# Supplementary material for: Modified McKenzie procedure for the treatment of fixed painful torticollis
Source: Neurosurg Focus Video. 2020 Oct 1;3(2):V10. doi: 10.3171/2020.5.FOCVID205 (PMC9542593; doi:10.3171/2020.5.FOCVID205)
Supplement: Supplemental Figure [file SupplementalFig1_FOCVID20-5.pdf]

NEUROSURGICAL FOCUS VIDEO

ONLINE ONLY

Supplemental material

Modified McKenzie procedure for the treatment of fixed painful torticollis

Aljuboori et al.

https://thejns.org/doi/abs/10.3171/2020.5.FOCVID205

DISCLAIMER The *Journal of Neurosurgery* acknowledges that the following section is published verbatim as submitted by the authors and did not go through either the *Journal's* peer-review or editing process.

© 2020 The authors, CC BY 4.0 (http://creativecommons.org/licenses/by/4.0/)

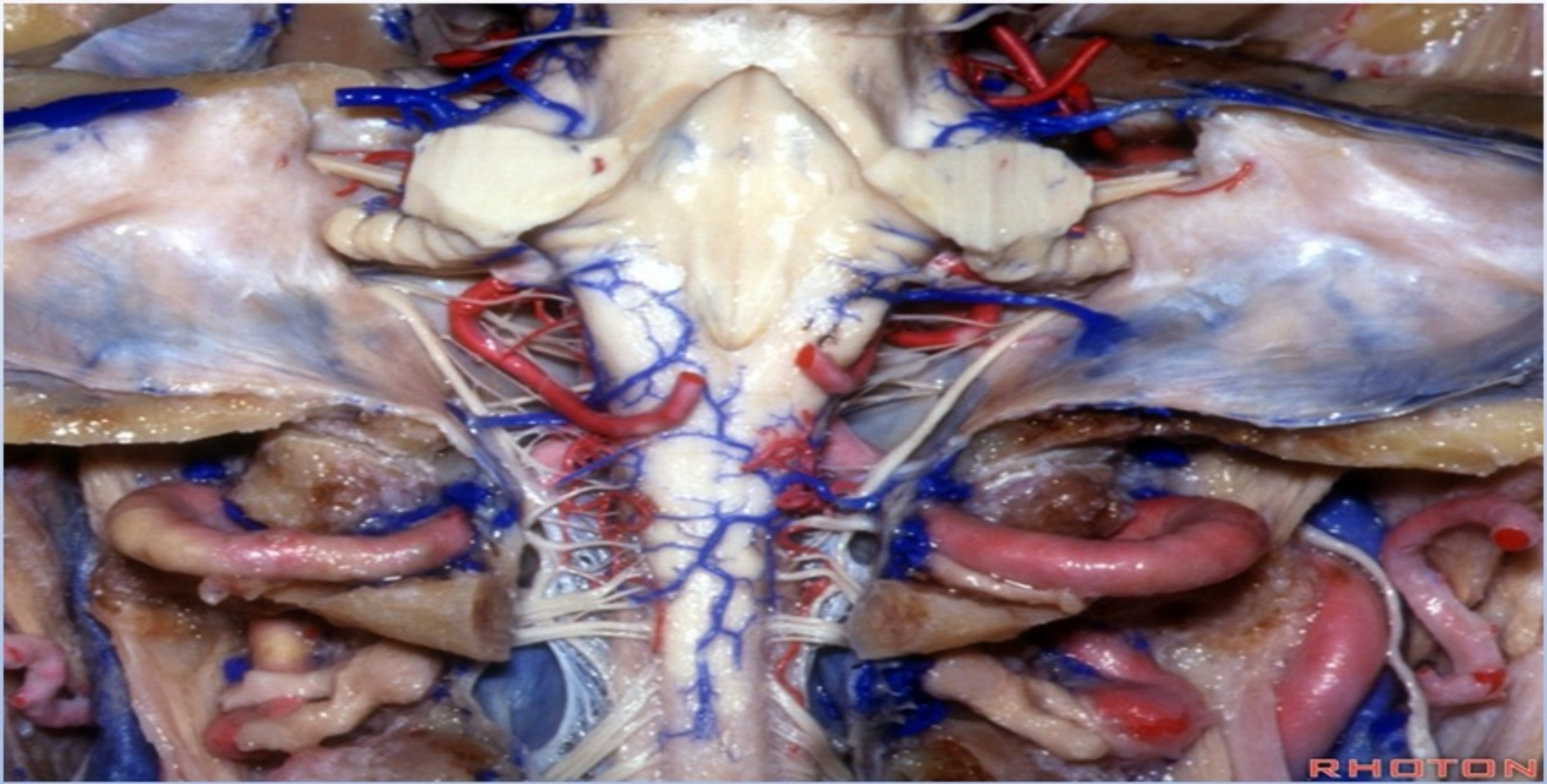

Fig. 1 A cadaveric dissection shows the posterior fossa, dorsal brainstem and spinal cord, cranial nerves, and related vascular structures. (Courtesy of the Rhoton Collection, American Association of Neurological Surgeons [AANS]/Neurosurgical Research and Education Foundation [NREF]).
